# ABCG8 Gene Responses to 8 Weeks Treadmill Running With or Without Pistachia atlantica (Baneh) Extraction in Female Rats

**DOI:** 10.5812/ijem.5305

**Published:** 2012-09-30

**Authors:** Abbass Ghanbari-Niaki, Saleh Rahmati- Ahmadabad, Navabeh Zare- Kookandeh

**Affiliations:** 1 Department of Physical Education and Sport Science, University of Mazandaran, Baboulsar, IR Iran

**Keywords:** Treadmill Running, ABCG8, Pistachia Atlantica, Female, Rat

## Abstract

**Background:**

It is well established that the excess cellular cholesterol concentration, as well as high density lipoprotein (HDL) and total cholesterol levels are strongly correlated with the incidence of coronary artery disease (CAD). Reverse cholesterol transport (RCT) is a term used to describe the efflux of excess cellular cholesterol. ABCG8 is a member of ABCG family that play a critical role in this process.

**Objectives:**

The current study was conducted to investigate the effect of endurance exercise with or without Pistachia atlantica (Baneh) extraction on small intestine and kidney ABCG8 gene, also plasma high density lipoprotein (HDL-c), triglyceride (TG), total cholesterol (TC), glucose, and estrogen levels in female rats.

**Materials and Methods:**

In this study twenty Wistar female rats (six to eight weeks old, 125-135 g weight) were used. Animals were randomly assigned into training (n = 10) and control (n = 10) groups and further divided into saline-control (SC), saline-training (ST), Baneh-control (BC), and Baneh-training (BT) groups. Training groups was given exercise on a motor-driven treadmill at 25 m/min (0% grade) for 60 min/day, 5 days/week for eight weeks. Animals were fed orally with Baneh extraction and saline for four week. After the last training session, rats were sacrificed, small intestine and kidney were excised, and ABCG8 expression was detected by Real-time PCR method. Plasma also was collected for plasma variable measurements. Statistical analysis was performed using a one way analysis of variance, and significance was accepted at P < 0.05. Correlation was calculated using the Pearson Product Moment correlation.

**Results:**

Exercise increased (P < 0.01) and Baneh reduced intestinal ABCG8 mRNA (P < 0.05). In kidney tissue, there wasn’t significant change between the groups (P < 0.40). Plasma HDL-C level was increased by exercise (P < 0.05) and decreased by Baneh (P < 0.02) that was correlated by intestine ABCG8 (r = 0.81, P < 0.001). Plasma TG and TC were unchanged, but glucose and estradiol were increased and decreased in Baneh groups (P < 0.02), respectively.

**Conclusions:**

Our study shows that exercise increases intestinal ABCG8 mRNA, and Baneh can increase plasma glucose concentration and reduce ABCG8 expression, HDL-C, and estrogen levels probably due to high fatty acid components.

## 1. Background

The process of reverse cholesterol transport (RCT) has well been documented that play an important role in whole body cholesterol metabolism and diminishing fat/lipid induced cardiovascular diseases, particularly atherosclerosis. It has also been reported that ATP-binding cassette (ABC) transporter family plays a crucial role as an initiator of RCT process (1, 2). The mammalian ATP-binding cassette (ABC) transporters consist of 49 individual transport proteins belonging to seven sub-families (ABCA to ABCG) involved in large number of functionally diverse trans-membrane proteins. ABC transporters are the main elements of RCT process which is crucial for cholesterol homeostasis, metabolism, and HDL formation (1, 2). The growing researches have shown the role of ABCA family, particularly ABCA1 as an initiator of RCT process in cholesterol efflux from different tissues (3-5). However, other family members of ABCs were neglected in this issue. Recently, some investigators focused on the role of ABCG subfamily members (expect G2) which exhibit different actions in transport of various materials such as amino acids, organic particles, peptides, sugars, lipopolysaccharides, several drugs , proteins, and cholesterol efflux (4, 6). It should be noted that most of studies have focused on the role of ABCG1 member and the role of other family members have been neglected in cholesterol efflux (4, 6). Recently some studies have been conducted by researchers to clarify the actions of ABCG8 in biliary cholesterol transport and its gene expression in different tissues such as liver and small intestine (2, 3). It has been postulated that expression of ABC family members could be affected by several factors such a nutrients, food conditions, and physical stress (7). It has been reported that administration of silymarin (1% and 3%) as dietary supplement to a high-cholesterol diet (HCD) reduced cholesterol absorption and rather restored the suppression of liver ABCG8, ABCG5 gene expression, and HDL concentration in rats fed by HCD diet (7, 8). Ven Den Bosch et al.reported that short periods of fasting increased expression of ABCA1 and ABCG8 in murine small intestine (9). Ghanbari-Niaki et al. reported that an endurance exercise training for 6 weeks (25 m/min , 90 min/ day, and five days/week) enhanced ABCA1 gene expression in rat liver and also increased plasma HDL-C, Pre β HDL, and lecithin cholesterol acyltransferase (LCAT) concentrations (7). Khabazian et al. observed a higher ABCA1 mRNA expression in rat small intestine following a 12 weeks endurance exercise training (25 m/min, 60min/ day, and five days / week) (8). It has been shown that eight weeks of low-intensity exercise (walking) significantly up-regulated ATP-binding cassette transporters A1 and G1 (i.e. ABCA1 and ABCG1, respectively) (9). On the other hand, Pistachia atlantica (Baneh) as a member of Anacardiaceae family has been reported to be rich in essential fatty acids and antioxidants being found in nuts. (13). It has also been measured that total amount of essential oils obtained from Pistachia atlantica was higher than that from any other Pistachia species (10). However, on the basis of our knowledge, there is no clear-cut information in relation to the effects of physical exercise and liquid extraction of Pistachia atlantica (Baneh) on ABCG8 gene expression in rat tissues.

## 2. Objectives

The current study was conducted to investigate the effects of an eight weeks treadmill running with our without oral administration of Pistachia atlantica extraction on small intestine and kidney ABCG8 gene expression in female rats.

## 3. Materials and Methods

### 3.1. Plant Material

The ripped fruit of Pistachia atlantica (Baneh) were collected from the fields of Maibod in Yazd province of Iran, dried in air for several days, and stored at –18 ° C until the use. Plant Material was identified by herbarium collection in department of biology, faculty of science, university of Mazandaran, Iran.

### 3.2. Preparation of the Pistachia atlantica (Baneh) Extraction

The extraction was prepared according to the Hamdan. et al. (15). Briefly, the whole ripped and dried fruit of Pistachia atlantica (Baneh) (10 g) was coarsely powdered and mixed with 150 ml of tap water, boiled for 45 min, and then cooled at room temperature. After the cooling, the mixture was filtered twice using a Watman filter (No. 4 filter). The volume of filtered solution was increased to 100 ml with tap water so that 1 ml was equivalent to 100 mg of starting material (11). Noteworthy that we did not use distilled water on the basis of herbalist’s recommendation. A fresh extraction was orally given at the dose of 100 mg/kg (7.5 ul/g of body weight) immediately after training session. The control groups have been treated at the same method and volume.

### 3.3. Preparation of the GC/MS Analyses

The whole ripped and dried fruits of Pistachia atlantica were grounded in house electronic grinder to a fine powder, part of which macerated by n-hexane (Merck Co., USA) for 72h at room temperature, extracted by soxhlet, and evaporated using a rotary evaporator. Chromatographic analysis was carried out on HP devices, 6890 series GCMS apparatus combined with a mass selective detector. The capillary column used was a HP-1MS. Helium was used as carrier gas. The fatty acid components of Pistachia atlantica extracts were determined using library search software from Wiley/NBS Registry Mass Spectral Data and in house “BASER Library of Fatty Acid Constituents”.

### 3.4. Animals

All experiment involving animals were conducted according to the policy of Iranian convention for the protection of vertebrate animals used for experimental and other scientific purposes; the protocol was approved by the Ethics Committee of the Sciences, University of Mazandaran (UMZ) and Babol University of Medical Sciences (BUMS, Mazandaran, Iran). Twenty Wistar female rats (6-8 weeks old, 125-135 g weight) were acquired from Pasteur’s Institute (Amol, Mazandaran) and maintained in the Central Animal House of Faculty of Physical Education and Sports Science of UMZ. Five rats were housed per cage (46-L volume) with a 12-hour: 12-hour light-dark cycle. Temperature and humidity were maintained at 22°C ± 1.4°C and 55.6% ± 4.0%, respectively. Diets (pellet form) and water were provided ad libitum. Animals were randomly assigned into control (n = 10) and training (n = 10) groups. Rats were further divided into saline-control (SC), saline-training (ST), Baneh-control (BC), and Baneh-training (BT) groups. The control groups remained sedentary, whereas the training groups underwent a moderate running exercise program.

### 3.5. Exercise Training Protocol

At first, the animals were familiarized with the rat treadmill apparatus, each day and for 4 days . A 14-lane motorized-driven treadmill was designed by the primary author (UMZ, Babolsar, Mazandaran, Iran). The exercise groups were trained for 8 weeks on a motor driven treadmill as previously reported elsewhere (7, 8). The rats were submitted to run at 25 m/min for 60 minutes, 5 d/week. The animals were killed 72 hours after the last exercise session. Food but not water was removed from the cages 3hours before the sacrifice.

### 3.6. Tissue Biopsies

Seventy-two hours after the last training session, rats were anesthetized with intra peritoneal administration of a mixture of ketamine (30– 50 mg / kg body weight) and xylazine (3– 5 mg / kg body weight). Small intestine and kidney were excised, cleaned, divided into two pieces, washed in ice-cold saline, and immediately frozen in liquid nitrogen and stored at − 80 ^°^ C until RNA extraction. Also blood was collected in EDAT test tubes as anticoagulant and immediately processed for plasma preparation, during 10min centrifugation at 3000rpm. Plasma was stored at -80C too, for future analysis.

### 3.7. RNA Isolation, cDNA Synthesis and Real-Time PCR

Total RNA was extracted from 80 to 100 mg of tissue using RNA purification kits (Accu Zol, Bioneer company). Complementary DNA (cDNA) was extended from oligo- (dt)_18_ primers (0.25 μg per reaction) using cDNA synthesis kit (AccuPower RT PreMix) according to the manufacturer’s instructions. cDNA concentration was 1 to 2 ng/25μl reaction. Real-time PCR was performed on light Cycler apparatus (Corbet). Real-time quantitative PCR was performed using QuantiFast SYBR Green PCR Kit (Cat. No. 204052; Qiagen, GmbH, Germany) in. ABCG8 sense primer was 5׳-CGTCAGATTTCCAATGACTTCCG-3׳ and antisense primer was 5׳-TCCGTCCTCCAGTTCATAGTACA-3׳ (AF351785, 241 bp) (12). The β-actin sense and antisense primers were 5׳-TATCGGCAATGAGCGGTTCC-3׳ and 5׳- CACTGTGTTGGCATAGAGG-3׳ (NM_031144 ,145 bp), respectively which were used as normalizer genes (13).

### 3.8. Plasma Lipid and Lipoprotein Concentrations

Plasma high density lipoprotein cholesterol (HDL) was determined by direct Immuno method (HDL-C Immuno FS, Pars Azmoun, Tehran, Iran); intra-assay coefficient of variation and sensitivity of the method were 1.2% and 0.03 mmol/l, resectively. Plasma total Triglyceride (TG) was determined by enzymatic (GPO, Glycerol-3-Phosphate Oxidase) colorimetric method (Pars Azmoun, Tehran, Iran); intra-assay coefficient of variation and sensitivity of the method were 2.2% and 1 mg/dL, respectively. Plasma total cholesterol (TC) was determined by enzymatic (CHOD-PAP, Cholesterol Oxidase-Amino Antipyrine) colorimetric method (Pars Azmoun, Tehran, Iran); intra-assay coefficient of variation and sensitivity of the method were 1.9% and 0.08 mmol/L, respectively. Estrogen concentration was determined by ELISA (Canada Inc, Ontario, Canada Estradiol ELISA, Diagnostics Biochem, 10 Pg/mL sensitivity).

### 3.9. Statistical Analysis

The data were analyzed by using a comparative threshold cycle method (CT). CT for each sample determined by implying a Rotor-Gene 3000 Software. Briefly, Δ-CT value was calculated by subtracting CT of ABCG8 gene from CT of β-actin. The ΔΔ-CT was calculated by subtracting Δ-CT (sample) values from Δ-CT (control). The relative quantification was then calculated by the expression 2^-ΔΔCT^ (14). The Kolmogorov-Smirnov test was used to determine normality of distribution, and variables were found to be normally distributed. All results were expressed as means ± SEM. Statistical analysis were performed using a one way analysis of variance. Least significant difference post hoc test was used in the event of a significant (P < .05) F ratio. Correlation was calculated using the Pearson Product Moment correlation. All statistical analysis was performed with SPSS (Version 13; SPSS, Chicago, IL).

## 4. Results

**Figure 1:**
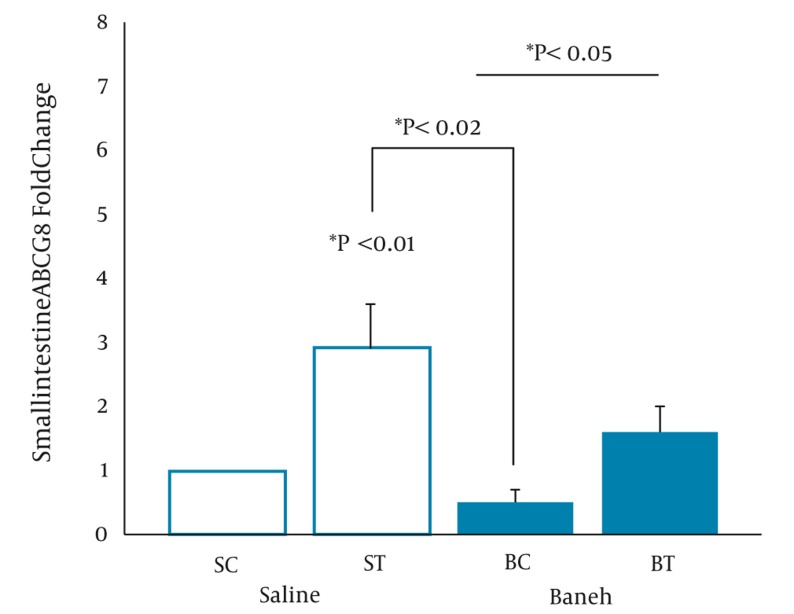
Real-Time PCR of Small Intestine ABCG8 Relative mRNA Expression in Saline- Control (SC), Saline-Training (ST), Baneh-Control (BC), and Baneh-Training (BT) Wild-Type Female Rats. Wild-type female rats Data expressed as mean ± SEM. Each column is for each group including 5 rats.

**Figure 2:**
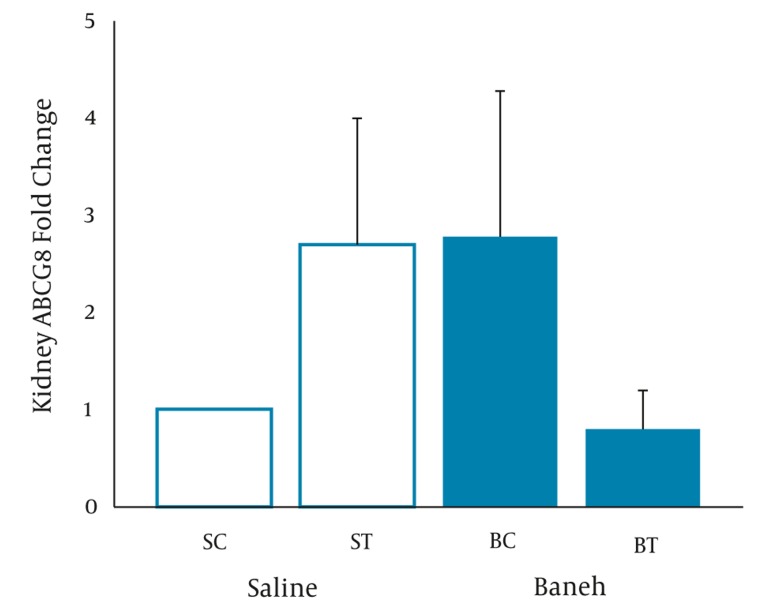
Real-Time PCR of Kidney ABCG8 Relative mRNA Expression in Saline- Control (SC), Saline-Training (ST), Baneh-Control (BC), and Baneh- Training (BT) Wild-Type Female Rats Wild-type female rats Data expressed as mean ± SEM. Each column is for each group including 5 rats.

**Figure 3:**
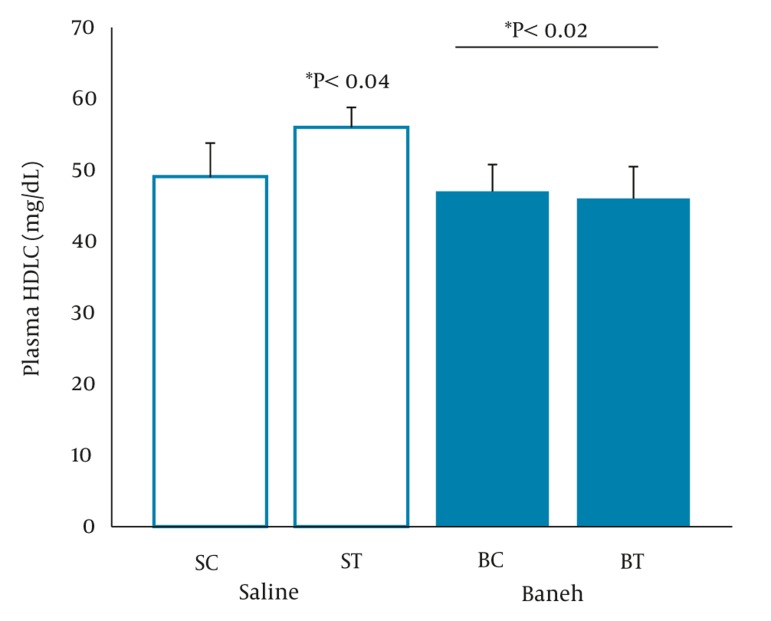
Plasma HDL-C Concentration (mg/dL) in Saline- Control (SC), Saline-Training (ST), Baneh-Control (BC), and Baneh-Training (BT) Wild- Type Female Rats. Wild-type female rats Data expressed as mean ± SEM. Each column is for each group including 5 rats.

**Figure 4:**
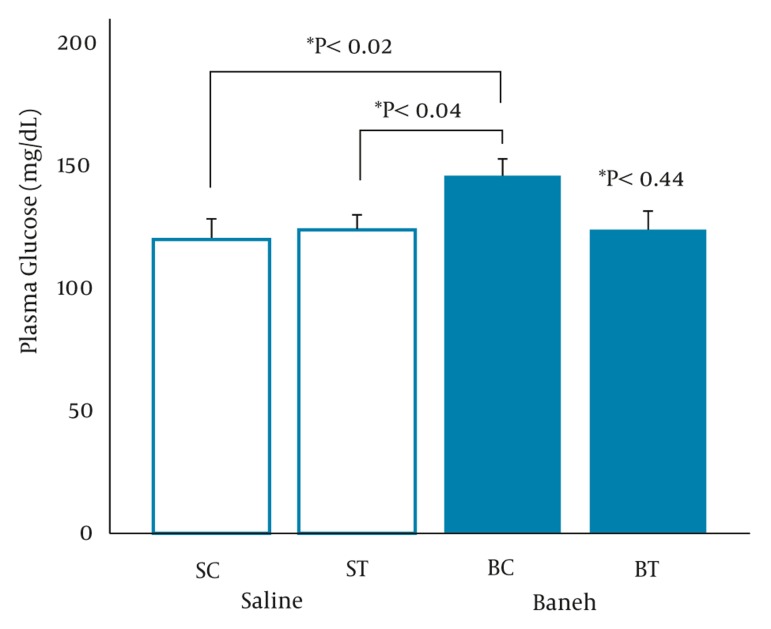
Plasma Glucose Concentration (mg/dL) in Saline- Control (SC), Saline-Training (ST), Baneh-Control (BC), and Baneh-Training (BT) Wild- Type Female Rats. Wild-type female rats Data expressed as mean ± SEM. Each column is for each group including 5 rats.

**Figure 5:**
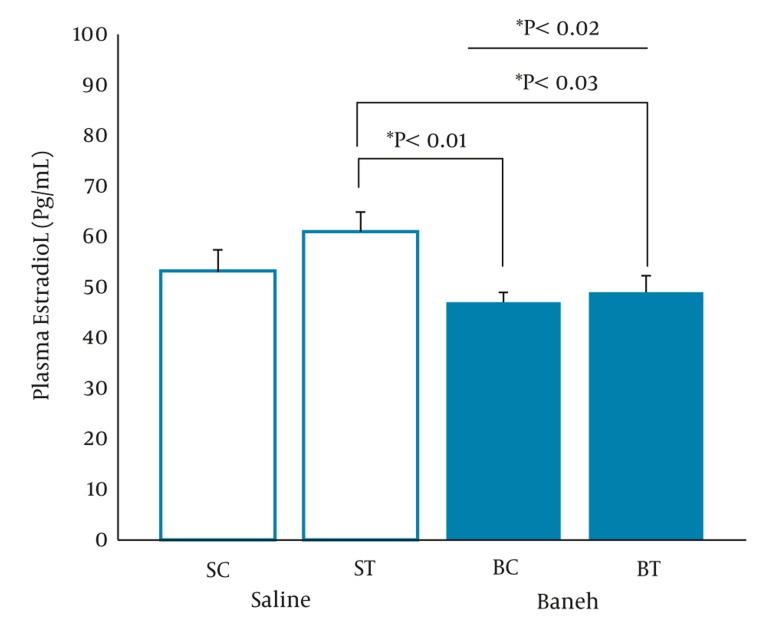
Plasma Estradiol Concentration (Pg/mL) in Saline- Control (SC), Saline-Training (ST), Baneh-Control (BC), and Baneh-Training (BT) Wild- Type Female Rats. Wild-type female rats Data expressed as mean ± SEM. Each column is for each group including 5 rats.

A significant difference in small intestine ABCG8 relative gene expression was found between all experimental groups (F = 5.096, P < 0.0.01) (Figure 1). Using a suitable post hoc test showed that the level of small intestine ABCG8 relative gene expression was higher in ST group compared to SC (P < 0.01), BC, and BT groups (P < 0.02) (Figure 1). In addition, a lower and higher ABCG8 gene expression have been found in Baneh-treated and exercised groups, respectively (Figure 1). No significant (F = 1.037, P < 0.0.40) differences were observed in the levels of kidney ABCG8 relative gene expression in all experimental animals (Figure 2). Using a one-way ANOVA revealed a significant difference in plasma HDL-C concentrations between the groups (F = 3.608, P < 0.0.03). A higher plasma HDL-C concentration was observed in ST group compared to SC group at the end of training program (P < 0.05). Data also indicated that Baneh-treated rats had lower and significant (P < 0.02) plasma HDL-C concentrations (Figure 3). Plasma glucose concentration was significantly higher in BC group compared to other groups (Figure 4). Using a one-way ANOVA revealed a significant (F = 3.06, P < 0.0.05) difference in plasma estradiol concentrations (Figure 5). It should be noted that the levels of plasma estradiol were lower in Baneh-treated rats compared to saline groups. However, a significant difference was also found between saline and Baneh-trained animals (Figure 5). Changes in plasma TC and TG concentrations were not significant between experimental groups (Table 1). A significant correlation was only found between tissue ABCG8 relative gene expression and plasma HDL-C concentrations but not in other measured variables (Table 2).

**Table 1:** Plasma Variables Concentration, in Saline- Control (SC), Saline-Training (ST), Baneh-Control (BC), and Baneh-Training (BT) Wild-Type Female Rats. Data expressed as mean ± SEM. Each column is for each group including 5 rats.

	SC	ST	BC	BT	F	*P* value
Plasma Cholesterol, mg/dL	85.6 ± 4.7	92.4 ± 6.8	86 ± 3.7	97 ± 6.4	0.096	0.43
Plasma Estradiol, Pg/mL	53 ± 4.4	61 ± 3.9	47 ± 2.03	49 ± 3.3	3.06	0.0.05
Plasma Glucose, mg/dL	120.6 ± 7.8	124.2 ± 5.8	146 ± 6.8	124.2 ± 7.5	2.70	0.08
Plasma Triglyceride, mg/dL	77 ± 4.01	84 ± 8.3	94 ± 7.8	86 ± 7.3	1.00	0.41
Plasma HDL-C, mg/dL	49 ± 4.8	56 ± 2.8	47 ± 3.8	46 ± 4.5	3.608	0.0.03

**Table 2:** Correlation Between ABCG8 Expression (Intestine and Kidney) and Other Measured Variables

	Intestine ABCG8		Kidney ABCG8	
	r Value	*P* value	r Value	*P* value
Plasma Cholesterol, mg/dL	0.26	0.25	-0.10	0.64
Plasma Estradiol, Pg/mL	0.35	0.13	0.22	0.33
Plasma Glucose, mg/dL	-0.13	0.56	0.33	0.56
Plasma Triglyceride, mg/dL	0.10	0.66	0.19	0.41
Plasma HDL-C, mg/dL	0.81	0.001	0.40	0.07

## 5. Discussion

The main findings of the current study were a higher and lower ABCG8 relative gene expression in trained and Baneh – treated rats, respectively. Other major result was a lower ABCG8 gene expression in trained Baneh-treated animals. A significant correlation was found between tissue ABCG8 and plasma HDL-C concentrations. On the basis of our best knowledge, this is the first study to demonstrate the responses of tissue ABCG8 gene expression to an endurance treadmill running and Baneh extraction in female rats. Our results are fairly in agreement with previous reports in relation to liver and small intestine ABCG8 expression (3, 15). Graf et al. used a Northern blot analysis method to show the expression of ABCG5 and ABCG8 overlapped in the liver and small intestine and, to a lesser extent, in the colon, where both ABCG5 and ABCG8 were expressed at the apical membrane (16). In our study a lower ABCG8 relative expression in small intestine has been observed in Pistachia atlantica (Baneh) - treated animals and exercise also restored the effect of Baneh-induced reduction of ABCG8 gene expression to some extent. A reduction in tissue ABCG8 gene expression following administration of Pistachia atlantica (Baneh) liquid extraction might be due to the content of fatty acids in this extraction and the time of feeding (immediately after the completion of training session). Data collected by using a GC-MS has shown that our used material included main following compositions; hexadecenoic acid (7.52%), Palmitinic acid (28.86%), trans-Oleic acid (49.28%), and n-Octadecanoic acid (3.87%). Perhaps the existence of a higher trans oleic acid and Palmitinic acid contents were enough to act as a high fat liquid extraction to reduce ABCG8 expression. In this regard, It has been reported that the administration of silymarin (1% and 3%) as dietary supplement to a high-cholesterol diet (HCD) reduced cholesterol absorption and rather restored the suppression of the liver ABCG8 and ABCG5 gene expressions and HDL concentration in rats fed by HCD diet (7, 8). In the present study, treadmill running exercise enhanced ABCG8 relative mRNA gene expression in saline-trained small intestine and fairly restored a Baneh-induced suppression of ABCG8 gene expression. A trend of enhancement and reduction were also observed in saline- trained and baneh-trained kidney, respectively. The role of ABCA1 in cholesterol metabolism has been well documented and recently the action of ABCG family such as ABCG1 and ABCG8 members in cholesterol homeostasis also received more attentions (17, 18). Considering the effect of physical exercise on ABC family gene expression, particularly ABCG members, there are very limited information. However, Ghanbari-Niaki et al. and Khabazian et al. reported a higher ABCA1 mRNA expression in trained rat liver and small intestine at the end of a treadmill running and an endurance exercise training (10, 11). They also reported that higher ABCA1 gene expressions were accompanied with a higher plasma HDL-C, Pre β HDL, and lecithin cholesterol acyltransferase (LCAT) concentrations. In one study on human subjects, a low-intensity exercise (walking, 10,000 steps /session and 3times /week) for 8weeks resulted in a significant and higher level of ABCA1 and ABCG1 gene expression in leukocytes (12). It has been suggested that four out of five mammalian ABCG members, ABCG1, ABCG4, ABCG5, ABCG8 have shown to play a considerable role in transporting sterols across membranes (19). Thus, considering the response of ABCG8 gene expression to a physical exercise, it could be suggested that ABCG8 possibly might follows the same pattern of ABCG1 response. The mechanism (s) by which the exercise training and Baneh treatment can influence the levels of ABCG8 gene expression in small intestine and kidney is poorly understood. However, several possible mechanisms could be considered. It has been suggested that a negative energy balance such as under a short-term fasting period has shown to increase ABCA1 and ABCG8 gene expression in murine small intestine (8). On the other hand, administration of high fat and high cholesterol diets have been observed to result in a reduction in ABCA1, ABCG4, and ABCG8 gene expression (20). The suppressive effect of unsaturated fatty acids on ABCA1 and ABCG1 gene expression in macrophage was also reported by Ku et al. (25). Ku et al. also focused on the role of liver X receptor in relation to the effect of saturated and unsaturated fatty acis on ABCA1 and ABCG1 mRNA expression. They found that with our without liver X receptor agonist unsaturated fatty acid not saturated fatty acid would reduce ABCA1 and ABCG mRNA expression (25). These events related to ABCA expression might be due to histone deacetylation which may play a role in repression of gene expression (25). It is also possible that post-transcriptionally, unsaturated fatty acids may facilitate ABCA1 protein degradation, which may involve in phosphorylation of ABCA1 by protein kinases (21). Using a GC-MS method by other researchers has provided a useful information about chemical compositions of Pistachia atlantica var. Mutica with high content of essential fatty acids (27). The total amount of essential oil obtained was 22% v/w which was higher than any other species of the genus Pistachia (10). Several study has shown that estrogen can be changed by exercise (22-25) and oil seed (26, 27). However, Our GC-MS data somehow confirm the previous reports with a little difference. In our study, main compositions of the bene sample were elaidic acids (Trans – delta oleic acid, 49.28%), palmitic acid (28.86%), and oleic acid (7.52%). Thus a reduction in ABCG8 gene expression after the administration of Baneh extraction might be explained by high content of elaidic acid in our Baneh extraction. It is also possible that a relatively long term given liquid extraction to rats might act as a high fat diet that could result in suppression of small intestine and kidney ABCG8 gene expression. Although we did not measure LXR’s receptor and peroxisome proliferator-activated receptors (PPARs), but any possible positive and negative change in these nuclear receptors might be involved in plasma lipid profiles, fatty acid, and cholesterol homeostasis as reported by us and some investigators and (12, 24). In summary, the results of present study indicate that treadmill running and orally given Baneh extraction independently affected on ABCG8 gene expression and plasma HDL-C concentrations. Data also indicate that small intestine and kidney ABCG8 did not response to treadmill running in similar manner, but their responses to Baneh administration were similar. The current results are also suggesting that orally given Baneh extraction in our experimental condition would increase plasma glucose and decrease plasma estrogen concentrations. Further studies are needed to clarify the effect of different doses of Baneh extraction on ABCG8 and other ABCG family members combined with treadmill running program.
